# Health risk assessment of heavy metal concentration in muscle of *Chelon auratus* and *Chelon saliens* from the southern Caspian Sea

**DOI:** 10.1007/s10653-022-01401-x

**Published:** 2022-11-02

**Authors:** Shima Bakhshalizadeh, Adeleh Rostamzadeh Liyafoyi, Francesco Fazio, Rafael Mora-Medina, Nahúm Ayala-Soldado

**Affiliations:** 1 Department of Marine Science, Caspian Sea Basin Research Center, University of Guilan, Rasht, Iran; 2 Department of Fisheries, Faculty of Natural Resources, University of Tehran, Karaj, Iran; 3 Department of Veterinary Science, Polo Universitario Dell’Annunziata, University of Messina, Messina, Italy; 4 Department of Anatomy and Comparative Pathology and Toxicology, Faculty of Veterinary Medicine, University of Córdoba, Córdoba, Spain

**Keywords:** Environmental monitoring, Food safety, Heavy metals pollution, Human health hazard

## Abstract

Heavy metals are one of the most serious pollutants in aquatic ecosystems, and their accumulation in fish products causes harmful effects on human health. In this context, we set out to determine the concentrations of heavy metals in the muscle of two fish species of commercial interest, *Chelon auratus* and *Chelon saliens* on the south coast of the Caspian Sea. We aimed to assess the degree of environment contamination in this area and to estimate the potential risk to human health derived from the consumption of fish. The mean concentrations of the different metals analysed were very varied in both species. In fact, some concentrations exceeded the permissible limits for the protection of human health for Cd and Pb, and some values of As were above those referenced by other authors in same species in the Caspian Sea. None of the estimated daily intake values exceeded the tolerable intake based on the consumption under consideration. Nonetheless, the accumulative hazard values evidenced a potential risk to human health, Pb and Hg being those giving a higher target hazard quotient. The cancer risk from exposure to As from fish consumption in children was above the “acceptable” risk to life. Thus, in view of the accumulative nature of heavy metals, a moderate and non-abusive fish consumption in this area, particularly in children, would be recommendable.

## Introduction

Over the past few decades, fish consumption has considerably increased as this food is an important source of proteins of a high biological value, some vitamins and minerals, and omega-3 polyunsaturated fatty acids (Bosch et al., 2016; Copat et al., 2018). Despite the benefits derived from its consumption, fish products can accumulate different pollutants, consequently triggering harmful effects on human health (Mozaffarian & Rimm, 2006). These pollutants sometimes exceed the limits permitted by the different legislations, although this does not always represent a human health hazard (Copat et al., 2013).

Due to their anthropogenic activity and their natural emissions, heavy metals are recognized as being one of the most serious pollutants in aquatic ecosystems, and they are causing concern worldwide. That concern stems from their intrinsic properties, such as their toxicity, persistence in the environment, non-degradability, bioaccumulative capacity and high potential for accessing and biomagnifying throughout the food chain (Cai et al., 2019; Häder et al., 2020; Pan et al., 2016; Zhong et al., 2018).

The Caspian Sea, with a surface of approximately 370,000 km^2^, is the largest inland sea in the world. This sea and its coastal areas are considered to be one of the world’s most valuable ecosystems due to their unique biodiversity (Lattuada et al., 2019). The alternation of shallow areas with different depressions, together with a wide range of salinities, provides it with a huge variety of biological niches with different depressions, giving rise to a great diversity of species (Bastami et al., 2017). Unfortunately, in recent years, the health of this ecosystem has been weakened, mainly due to anthropogenic pressures like, for example, the extraction of petrol and mining activity (Lattuada et al., 2019; Poorbagher et al., 2017).

Very many scientific articles have used fish as bioindicators of environmental pollution (Naigaga et al., 2011; Authman et al., 2015; Vaseem & Banerjee, 2016; Plessl et al., 2017; Łuczyńska et al., 2018). In addition, Yancheva et al. (2015) reported in their research that muscle tissue was most appropriate for the evaluation of human health risks. In this respect, we set out to determine the concentrations of heavy metals in the muscle of two fish species of commercial interest, *Chelon auratus* and *Chelon saliens*, on the south coast of the Caspian Sea, with two main objectives: to assess the degree of environment contamination in this area; and to estimate the potential risk to human health derived from the consumption of this fish.

## Material and methods

A total of 49 fish from two different species, 20 *Chelon auratus* and 29 *Chelon saliens*, were obtained randomly in the southern area of the Caspian Sea. Before taking samples, the animals were cleaned with distilled water to remove any dirt or possible external substances that might pollute them. Muscle samples were taken from each individual for the determination of the metals, with each muscle sample weighing 1 g. The instrument used to cut the muscle tissue was previously washed with 1% nitric acid. The sampling was carried out in accordance with the European protection rules for animals used for scientific purposes (Directive 2010/63).

The heavy metals analysed were as follows: Al, As, Cd, Co, Cr, Cu, Fe, Hg, Mn, Ni, Pb, Sn, Tl, V and Zn. For their quantification, an inductively coupled plasma mass spectrometer (ICP-MS) was used. With respect to the preparation of the samples for their analysis, each sample, individually, was homogenized and digested in a digestion solution with 15 ml of 65% nitric acid (HNO_3_) and hydrogen peroxide heated on a plate at 200 ºC. When the sample reached a volume of 5 mL, before drying it and after cooling, the content was decanted into Falcon tubes and diluted in deionized water (Milli-Q Millipore 18.2 MΩ / cm of resistivity) until reaching 30 mL. The analytical blanks were processed in the same way, and the concentrations were determined using standard solutions prepared in the same acid matrix.

### Metal pollution index

The Metal Pollution Index (MPI) was calculated to indicate the total content of metal elements accumulated in the muscles sampled in both species For this purpose, the following equation was applied (Usero et al., 2005):

$$\mathrm{MPI} = {(Cf1 \times Cf2\dots Cfn)}^{1/n}$$

where Cf _*n*_ is equal to the concentration of the metal *n* in the sample.

### Evaluation of human health risk

To estimate the potential risk to human health from fish consumption, the following indicators were calculated: the Estimated Daily Intake (EDI), comparing it with Tolerable Daily Intake (TDI) recommended by the joint Food and Agriculture Organization/World Health Organization (FAO/WHO) Expert Committee on Food Additives (JECFA); the Target Hazard Quotient (THQ), with the aim of evaluating any possible warnings with regard to adverse effects and Cancer Risk (CR) for As.

### Estimated daily intake

All the risk limits and factors were calculated for adults by assuming the mean daily world intake (IR) of fish *per capita* of 40 g/day (FAOSTAT, 2022), and a body weight (BW) of 70 kg (USEPA, 2000). For children (between 3 and 6 years), the IR was established at 20 g/day and a BW of 14.5 kg.

The EDI (µg/g/day) was calculated by using the following formula:

$$\mathrm{EDI}=C\times \mathrm{IR}/\mathrm{BW}$$

where *C* is the concentration of metal (mg/kg wet weight), IR is the mean daily intake and BW is the body weight.

### Non-carcinogenic risk and carcinogenic risk

The risk factors were reckoned by basing them on the directives of the United States Environmental Protection Agency (USEPA) (USEPA, 2000). In accordance with this guide, we took for granted that the dose ingested was equal to that of the pollutant absorbed, and that cooking did not have any effect on the concentration of heavy metals (USEPA, 1989; Cooper et al., 1991). Also, As can be found in the environment and in living beings in two forms, both organic and inorganic As. The latter is the most toxic form and its percentage over total As is highly variable depending on the type of food (EFSA, 2009; Jomova & Valko, 2011). The same as Copat et al. (2013), to calculate the equations, we assumed that inorganic As represented 3% of the total.

The non-carcinogenic risk was assessed by calculating the THQ. Indeed, the THQ values were evaluated for each heavy metal separately and were calculated as per the following equation:

$$\mathrm{THQ}=(C \times \mathrm{IR} \times \mathrm{EF} \times \mathrm{ED}) /(\mathrm{BW} \times \mathrm{AT}\times \mathrm{RfD})$$

When the THQ is over 1, i.e. higher than the reference dose, adverse effects may appear.

The accumulative risk to health was assessed by adding the THQ value of each metal, expressed as Total Target Hazard Quotient (TTHQ), as follows:

$$\mathrm{TTHQ} = \mathrm{THQ}1+\mathrm{THQ}2\dots \mathrm{THQ}n$$

The higher the TTHQ value, the higher the level of concern.

The CR was calculated only for As as it is the only metal that is cancerigenous orally. For this, the Oral Slope Factor (OSF) has been established at 1.5 mg/kg/day for that metal by USEPA (IRIS, 2022). If the CR is above the acceptable risk to life of 1 × 10^–5^, the value considered by the USEPA (2000), indicates a greater probability of 1 individual out of 100,000 developing cancer. The equation is expressed as follows (USEPA, 1989):

$$\mathrm{CR} = (\mathrm{EF} \times \mathrm{ED} \times \mathrm{IR} \times C \times \mathrm{OSF})/(\mathrm{BW} \times \mathrm{AT})$$

In the previous equations, EF is the exposure frequency (365 days/year); ED is the exposure duration (adults: 70 years; children: 6 years); IR is the mean daily intake (adults: 40 g/day; children: 20 g/day); C is the mean concentration of metal in fish (mg/kg wet weight); RfD is the reference oral dose (mg/kg/day); BW is the body weight (adults: 70 kg; children: 14.5 kg); AT is the average exposure time (it is equal to EF × ED), and OSF is the Oral Slope Factor (mg/kg/day). The RfD provided by the online database of the EPA’s Integrated Risk Information System (IRIS) (IRIS, 2022) for As, Cd, Cr, Hg, Mn, Ni, Sn, Tl, V and Zn, are 3 × 10^–4^; 1 × 10^–3^; 3 × 10^–3^; 1 × 10^–4^; 1.4 × 10^–1^; 2 × 10^–2^; 5 × 10^–3^, 8 × 10^–5^; 9 × 10^–3^ and 3 × 10^–1^, respectively. Regional Screening Levels (RSLs) from USEPA’s Generic Tables indicate an RfD of 3 × 10^–4^, 4 × 10^–2^ and 7 × 10^–1^ for Co, Cu and Fe, respectively (USEPA, 2021). The RfD of Pb is 5 × 10^–4^ (EFSA, 2010) and that of Al is 1.43 × 10^–1^, used by Zioła-Frankowska et al. (2021).

### Statistical analysis

The statistical analysis of the data was made using SAS/STAT Software (SAS Institute Inc., Cary, NC). A variance analysis (ANOVA) was performed on a non-parametric path in order to establish the statistical differences in the concentration of the different metals between species. A value of *P* < 0.05 was taken as being significant.

## Results and discussion

### Heavy metals concentrations

The medians, means and standard deviations of all the metals analysed are shown in Table 1. The statistical analysis of the concentrations revealed that only Cd presented statistically significant differences (*P* < 0.05) between species. To be specific, a higher concentration of this metal was found in the *Chelon saliens* samples. However, it could be seen that the concentrations of the different metals were very varied and gave from the highest to lowest concentrations in this order: Fe > Zn > Cu > Al > Mn > As > Ni > Pb > V > Cr > Co > Sn > Cd > Hg > Tl. The average ranges, without distinguishing between species were: Fe (0.001–71.562 mg/kg); Zn (1.025–33.645 mg/kg); Cu (0.701–9.921 mg/kg); Al (0.055–86.875 mg/kg); Mn (0.001–12.567 mg/kg); As (0.191–6.031 mg/kg); Ni (0.009–8.396 mg/kg); Pb (0.029–0.867 mg/kg);V (0.004–24.783 mg/kg); Cr (0.002–0.754 mg/kg); Co (0.015–3.500 mg/kg); Sn (0–0.923 mg/kg); Cd (0.012–0.835 mg/kg); Hg (0.002–0.139 mg/kg) and Tl (0–0.171 mg/kg).

**Table 1:** Median, mean concentrations (mg/kg w.w.) and standard deviations (SD) of analysed metals in *Chelon auratus* and *Chelon saliens*. Metal Pollution Index (MPI) for *Chelon auratus* and *Chelon saliens*

Metal (mg/kg w.w.)	*Chelon auratus*			*Chelon saliens*		
	Median	Mean	SD	Median	Mean	SD
Al	2.514	3.157	2.482	2.905	6.144	16.029
As	0.529	0.606	0.442	0.651	1.031	1.273
Cd^*^	0.035	0.077	0.110	0.093	0.153	0.181
Co	0.109	0.136	0.103	0.154	0.344	0.741
Cr	0.145	0.207	0.222	0.143	0.204	0.195
Cu	3.250	3.880	2.683	4.179	4.321	2.501
Fe	23.139	25.984	17.483	18.449	25.187	23.289
Hg	0.036	0.043	0.028	0.048	0.051	0.040
Mn	1.795	2.221	1.888	1.421	2.461	3.060
Ni	0.184	0.221	0.175	0.191	0.539	1.560
Pb	0.159	0.218	0.204	0.192	0.265	0.228
Sn	0.049	0.067	0.077	0	0.120	0.228
Tl	0.005	0.016	0.036	0.008	0.019	0.038
V	0.188	0.170	0.148	0.163	1.339	5.374
Zn	4.710	5.729	6.704	3.470	5.702	6.913
MPI		0.665			0.416	

*P < 0.05 between species

It is important to highlight that metals like Cr, Co, Cu, Fe, Ni, Mn, Sn and Zn are essential for physiological processes so they are normally present in the tissues at certain concentrations, and their total absence in the organisms causes irreversible and severe damage to the vital functions (Zoroddu et al., 2019). Nevertheless, these metals could trigger toxic effects if their exposure dose exceeds a certain level (Mertz, 1981). All the concentrations found for these metals were placed within the range of concentrations (mg/kg) previously found by Sheikhzadeh and Hamidian (2021) in the Caspian Sea: Cr (0.00–10.01), Co (0.00–1.34), Cu (0.00–160.39), Fe (2.1–1343.5), Mn (0.1–15.7), Ni (0.09–8.1), Zn (1.72–219.22).

On the other hand, metals like As, Cd, Hg and Pb are considered as being non-essential since they do not possess any known biological function (Zoroddu et al., 2019). Arsenic intake disables the function of enzymes, some significant anions and cations, transcriptional events in cells, and causes health effects (Raju, 2022). Cadmium exposure has been associated with nephrotoxicity, hepatotoxicity and effects on the immune system, bones and male reproductive physiology (Cannas et al., 2020). With regard to Hg, dietary consumption of marine fish and other seafood is the most common source of exposure to this metal (Cannas et al., 2020; Driscoll et al., 2013). All Hg forms exhibit toxicological characteristics including neurotoxicity, nephrotoxicity, and gastrointestinal toxicity (Valko et al., 2005). Pb has been shown to adversely affect the functions of hepatic, endocrine, reproductive and digestive systems (Charkiewicz & Backstrand, 2020).

The European Union, by means of Regulation (EC) 78/2005, only established a maximum content in fish for Cd, Hg and Pb, so the maximum contents for these metals have been fixed at 0.05, 0.5 and 0.2 mg/kg wet weight for Cd, Hg and Pb, respectively. Considering the concentration ranges obtained in our study for Cd (0.012–0.835 mg/kg) and Pb (0.029–0.867 mg/kg), it can be seen that several samples exceed the maximum content permitted by the European Union for these metals. In the case of Hg (0.002–0.139 mg/kg), in no way did it exceed the maximum content. Besides that, in the review made by Sheikhzadeh and Hamidian (2021), the range of concentrations observed in the Caspian Sea for Hg oscillated between 0.044 mg/kg (mean) in the whole body of *Cyprinus carpio* and up to 3.5 mg/kg in the muscle tissue of *Huso huso*. Thus, the range of concentrations determined for that metal is lower than that reported in other studies.

In our work, the mean As concentration found in *Chelon saliens* (1.03 mg/kg) was higher than that reported by Fathabad et al. (2021) (0.35 mg/kg) in *Chelon saliens* and (0.80 mg/kg) in *Cyprinus carpio,* in the Caspian Sea. These results are of great importance since chronic intoxication by As can cause serious diseases, including different types of cancer (Das et al., 2004).

### Metal pollution index

The MPI permitted us to present all the metal concentrations as a single value. According to Brady et al. (2015); when the MPI is between 2 and 3, this indicates a moderate pollution; when it is established at between 1 and 2 the pollution is mild; whereas, below 1 means that the pollution impact is insignificant. In our study, the MPI values were of 0.665 for *Chelon auratus* and of 0.416 for *Chelon saliens*, so we are able to affirm that the metals in this species were no significance (Table 1). In this context, the calculation of MPI in sediments or in filtering organisms, as reported by Usero et al. (2005), could be a source of interest for evaluating environmental pollution.

### Estimated daily intake

The EDI values for children and adults are presented in Table 2. Here, none of the values exceeded the tolerable intake suggested by the joint FAO/WHO Expert Committee on Food Additives (JEFCA) (WHO, 2022) for the metals for which this indicator is available. Thus, it can be said that there would be no risk to people’s health associated with the consumption of fish with those metal concentrations and at a portion of 40 and 20 g/day in adults and children, respectively. However, the EDI is dependent on metal concentrations, the size of the portion, and on the body weight. With that in mind, our calculations were based on the mean world consumption of fish, which is highly variable according to the region. For example, in the southeast Asia, the mean consumption of fish is very much higher (90 g/day) (FAOSTAT, 2022) than in some countries in Africa, such as Algeria, where fish is scarcely included in the diet (0.09 g/day) (FAOSTAT, 2022). That is why it is important to take into account these factors since they may directly affect the EDI.

**Table 2:** Estimated Daily Intake (EDI) (µg/kg/d) in adult and child in *Chelon auratus* and *Chelon saliens* compared with tolerable intake (µg/kg/d) suggested by Joint FAO/WHO Expert Committee on Food Additive (JECFA)

Tolerable intake (µg/kg/d)	Metal	*Chelon auratus*		*Chelon saliens*	
		EDI Child	EDI Adult	EDI Child	EDI Adult
1000	Al	8.474	3.511	4.355	1.804
15	As^a^	0.042	0.018	0.025	0.010
7	Cd	0.210	0.087	0.107	0.044
–	Co	0.474	0.197	0.188	0.078
–	Cr	0.280	0.116	0.286	0.118
500	Cu	5.959	2.469	5.352	2.217
800	Fe	34.740	14.392	35.840	14.848
4	Hg	0.070	0.029	0.059	0.024
–	Mn	3.349	1.406	3.064	1.269
–	Ni	0.743	0.307	0.305	0.126
25	Pb	0.365	0.151	0.300	0.124
–	Sn	0.165	0.069	0.092	0.038
–	Tl	0.026	0.011	0.022	0.009
–	V	1.847	0.765	0.234	0.097
300	Zn	7.864	3.258	7.903	3.274

^a^As calculations were made by assuming the inorganic As as being 3% of the total concentration

The THQ for children and for adults is given in Table 3. The values for Al, As, Cd, Cr, Cu, Fe, Hg, Mn, Ni, Pb, Sn, Tl, V and Zn are all under 1, which indicates that there would be no risk of developing adverse effects from the consumption of fish with those metal concentrations. Only the THQ of Co for children in *Chelon auratus* was higher than 1, being established at 1.582. In this sense, we have taken into account the RfD supplied by the USEPA, which has fixed 3 × 10^–4^ for this metal. It is essential to point out that a dose of 3 × 10^–4^ mg/day/kg BW of Co is equal to approximately 21 µg Co/day for an adult of 70 kg, which corresponds to the typical dietary intake of Co in the U.S.A. (5–40 µg Co/día). Therefore, the adoption of this value would suggest that a large part of the population exceeds a safe dose of Co simply due to traces of this metal being of a natural origin in its diet (Finley et al., 2012). For example, the directives of the European Food Safety Authority (EFSA) with regard to supplementation with Co have established a dose of 600 µg/day for a person of 60 kg, i.e., 1 × 10^–2^ mg/day/kg BW (EFSA, 2009). So, if we apply an assumed RfD of 1 × 10^–2^ mg/day/kg BW, the THQ calculated for the Co would be situated below 1 in all the cases.

**Table 3:** Target Hazard Quotient (THQ) and Total Target Hazard Quotient (TTHQ) for metals analysed in *Chelon auratus* and *Chelon saliens*

Metal	*Chelon auratus*		*Chelon saliens*	
	THQ Child	THQ Adult	THQ Child	THQ Adult
Al	0.059	0.025	0.030	0.013
As^a^	0.142	0.059	0.084	0.035
Cd	0.211	0.087	0.107	0.044
Co	1.582^*^	0.656	0.625	0.259
Cr	0.094	0.039	0.095	0.039
Cu	0.149	0.062	0.134	0.055
Fe	0.050	0.021	0.051	0.021
Hg	0.700	0.290	0.587	0.243
Mn	0.024	0.010	0.022	0.009
Ni	0.037	0.015	0.015	0.006
Pb	0.731	0.303	0.601	0.249
Sn	0.033	0.014	0.018	0.008
Tl	0.320	0.132	0.274	0.113
V	0.205	0.085	0.026	0.011
Zn	0.026	0.011	0.026	0.012
TTHQ	4.363	1.808	2.695	1.117

^*^Above 1

^a^As calculations were made by assuming the inorganic As as being 3% of the total concentration

Nevertheless, although all the THQs individualized per metals were established as being under the reference dose, it was observed that the accumulative risk to health values (TTHQ) obtained was higher than 1, and would therefore be a potential hazard to health through consuming this fish (Table 3). In this respect, the metals of most concern, without counting Co, were Pb and Hg as their THQ was higher.

The CR from As was established at 5.4 × 10^–6^ y 3.2 × 10^–6^ in adults for *Chelon auratus* and *Chelon saliens*, respectively. In children, the CR calculated was 2.65 × 10^–5^ and 1.56 × 10^–5^ for *Chelon auratus* and *Chelon saliens*, respectively. There is a need to specify a level of “Acceptable” Risk to Life (ARL) in order to calculate consumption limits and evaluate cancerigenous effects. Following the USEPA rules (USEPA, 2000), we considered it to be appropriate to use an ARL of 1 at 100,000 (10^–5^). Thus, in adults, the CR was under the ARL and considered to be insignificant. However, the values in children were over the ARL, indicating a greater probability of 1 individual out of 100,000 developing cancer due to exposure to As through fish consumption, which should be a cause for concern.

## Conclusions

The results obtained in this study showed ample mean concentrations of heavy metals both in *Chelon auratus*, and in *Chelon saliens*, with the highest levels recorded being of Fe, Zn, Cu, Al and Mn. In relation to the concentrations of non-essential metals, in the case of Cd and Pb some concentrations exceeded the maximum permissible limits for the protection of human health established by the European Commission for those elements. With respect to the mean concentration of As in *Chelon saliens*, the values found were above those referenced by other authors for the same species in the Caspian Sea. Also, although the MPI has indicated that pollution does not have any significant impact, given the dangers and toxicity at low concentrations that they represent, it is still important to highlight these findings since those levels of pollution are disturbing.

Although none of the EDI values exceeded the tolerable intake based on the consumption under consideration, body weight being a dependent factor in the calculation of the EDI, its values in children were higher and, consequently, they were nearer to the tolerable intake values established. However, the TTHQ values evidenced a potential risk to human health in consuming this fish, Pb and Hg being those giving a higher THQ.

The CR in children is placed above the ARL established by the USEPA, which infers a greater probability of developing cancer due to exposure to As from fish consumption, which ought to be a reason for concern. Thus, in view of the accumulative nature of heavy metals, it would be important to review the tolerable intake and limit of the eating of fish, and a moderate and non-abusive fish consumption in this area, particularly in children, would be recommendable.

In that respect, the results obtained have demonstrated the high bioaccumulation potential of heavy metals signifying adverse implications for human health, so it would be paramount to carry out extensive samplings both in fish (and other species) and in the environment (water and sediments), as well as recommending preventive measures directed towards limiting and/or reducing the excessive exposure of people to heavy metal contents.

## Data Availability

The data that support the findings of this study are available from the corresponding author.
